# Rapid controlled synthesis of gold–platinum nanorods with excellent photothermal properties under 808 nm excitation

**DOI:** 10.3762/bjnano.12.37

**Published:** 2021-05-17

**Authors:** Jialin Wang, Qianqian Duan, Min Yang, Boye Zhang, Li Guo, Pengcui Li, Wendong Zhang, Shengbo Sang

**Affiliations:** 1 MicroNano System Research Center, College of Information and Computer & Key Laboratory of Advanced Transducers and Intelligent Control System of Ministry of Education and Shanxi Province, Taiyuan University of Technology, Taiyuan 030024, Shanxi, China; 2 Department of Orthopedics, the Second Hospital of Shanxi Medical University, Taiyuan 030024, Shanxi, China

**Keywords:** AuNRs, local surface plasmon resonance (LSPR), photothermal conversion efficiency, photothermal transduction agents, platinum

## Abstract

Noble metal nanomaterials are particularly suitable as photothermal transduction agents (PTAs) with high photothermal conversion efficiency (PCE) due to local surface plasmon resonance (LSPR). Studies on different gold–platinum (Au–Pt) bimetal nanoparticles exhibiting the LSPR effect have provided a new idea for the synthesis of excellent PTAs. But there is no simple and scalable method for the controllable synthesis of Au–Pt nanoparticles with adjustable LSPR wavelength range. In this work, the effects of Ag^+^ and K_2_PtCl_4_ on the deposition of Pt on the surface of gold nanorods (AuNRs) were investigated. A fast, precise, and controlled synthesis of dumbbell-like Pt-coated AuNRs (Au@Pt NRs) under mild conditions is proposed. The synthesized Au@Pt NRs have a longitudinal LSPR wavelength of 812 nm, which is very close to a common laser wavelength of 808 nm. The Au@Pt NRs exhibit excellent photothermal properties when irradiated with a laser. The temperature increased by more than 36 °C after irradiation for 10 min, with a PCE of about 78.77%, which is much higher than that of AuNRs (57.33%). In addition, even after four on/off cycles, the Au@Pt NRs are able to maintain the photothermal properties and retain their optical properties, indicating that they have excellent photothermal stability and reusability.

## Introduction

On the surface of noble metal nanoparticles, when the wavelength of incident light resonates with the light absorption wavelength of the nanoparticles, a significant part of the photon energy is absorbed through LSPR [1–2]. The LSPR effect excites the free conduction band electrons and energy is released in the form of heat through nonradiative decay [3–4]. Au, Ag, Pt, and other noble metal nanoparticles all exhibit an obvious LSPR effect and strong spectral absorption in the UV–vis range. Hence, these nanoparticles can be applied as excellent PTAs. The PCE of PTAs is directly related to the light absorption capacity. For plasmon nanoparticles, their absorption will be significantly enhanced when the illumination laser wavelength is equal to their LSPR wavelength. In addition, the microstructure of metal nanomaterials, especially the aspect ratio (ratio between length and width), determines the position of the LSPR peak. Therefore, regulating the microstructure of nanomaterials is an effective means to control the LSPR wavelength.

Au nanomaterials, especially those with anisotropic structure, such as AuNRs, Au nanocages [5–6], Au nanostars [7–8], Au nanospheres [9–10], and Au nanoshells [11–12], show great potential as PTAs. Particularly, AuNRs, with a high inherent absorption–scattering ratio, simple and controllable synthesis, and high PCE [13–14], have become one of the most extensively researched PTAs. It is acknowledged that thermal stability is extremely critical for noble metal PTAs. The observed melting temperature reduction at the nanoscale will affect the integrity of structure and morphology, thus affecting the optical properties during heating [15–16]. Pt nanoparticles have better light and thermal stability then Au nanoparticles [17]. Au–Pt bimetal nanoparticles may not only further enrich the functions of nanostructures, but the spatial distribution of both elements also plays an important role in adjusting the properties.

Most of the reported Au–Pt bimetal nanoparticles are isotropic, such as alloys or core–shell structures [18–20]. However, the LSPR peaks of such alloys or core–shell structures are located in the visible light region and inhibit the plasma performance [21]. Significant efforts have been made to synthesize Au–Pt bimetal nanoparticles with LSPR bands in the near-infrared (NIR) region [22–27]. Feng et al. developed a simple room-temperature procedure to form rod-shaped Au@Pt nanostructures, where tiny Pt nanodots are distributed homogeneously on the surface of the AuNRs [25]. Rong et al. synthesized bimetal noncompact dendritic Pt shell-decorated AuNRs with spatial control of the Pt growth [26]. Grzelczak et al. developed two different deposition modes of Pt on the surface of AuNRs by removing or keeping the Ag^+^ ions used in the formation of AuNRs [27]. However, these studies have not yet clearly evaluated the photothermal properties of their products.

**Scheme 1:**
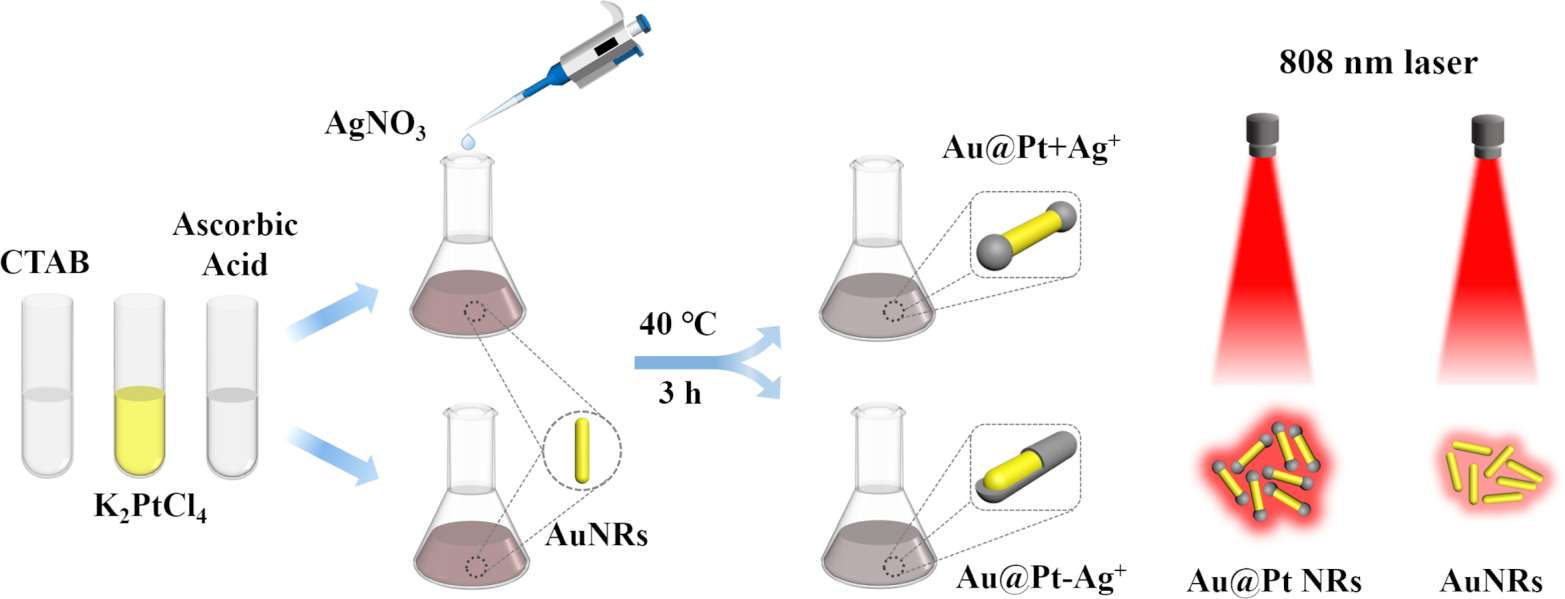
Schematic illustration of the synthesis and photothermal of the Au@Pt NRs.

The key factors to be considered when selecting noble metal nanoscale PTAs are the wavelength of the LSPR peak, PCE, and photothermal stability. Based on previous research results from a preliminary experiment of Pt deposition on AuNRs, it is impossible to determine the excessive unreacted Ag^+^ concentration in the freshly prepared AuNRs solution, which hinders the accurate control of the synthesis of Au@Pt NRs. Herein, we used AuNRs as seeds and re-added reagents in the reaction solution to study the influence of Ag^+^ and K_2_PtCl_4_ on the morphology and optical properties of the synthesized Au@Pt NRs. After continuous optimization of the synthesis process, a precise and controllable synthesis method of dumbbell-like Au@Pt NRs was proposed (Scheme 1). Au@Pt NRs with ideal size and structure formed after depositing Pt on the tip of AuNRs, and their LSPR peak can be adjusted to a desired range. Importantly, we evaluated that the Au@Pt NRs have high PCE and fairly great photothermal stability under 808 nm laser irradiation, which proves that they have great potential as an excellent PTA.

## Results and Discussion

### Synthesis and characterization of the AuNRs

**Figure 1:**
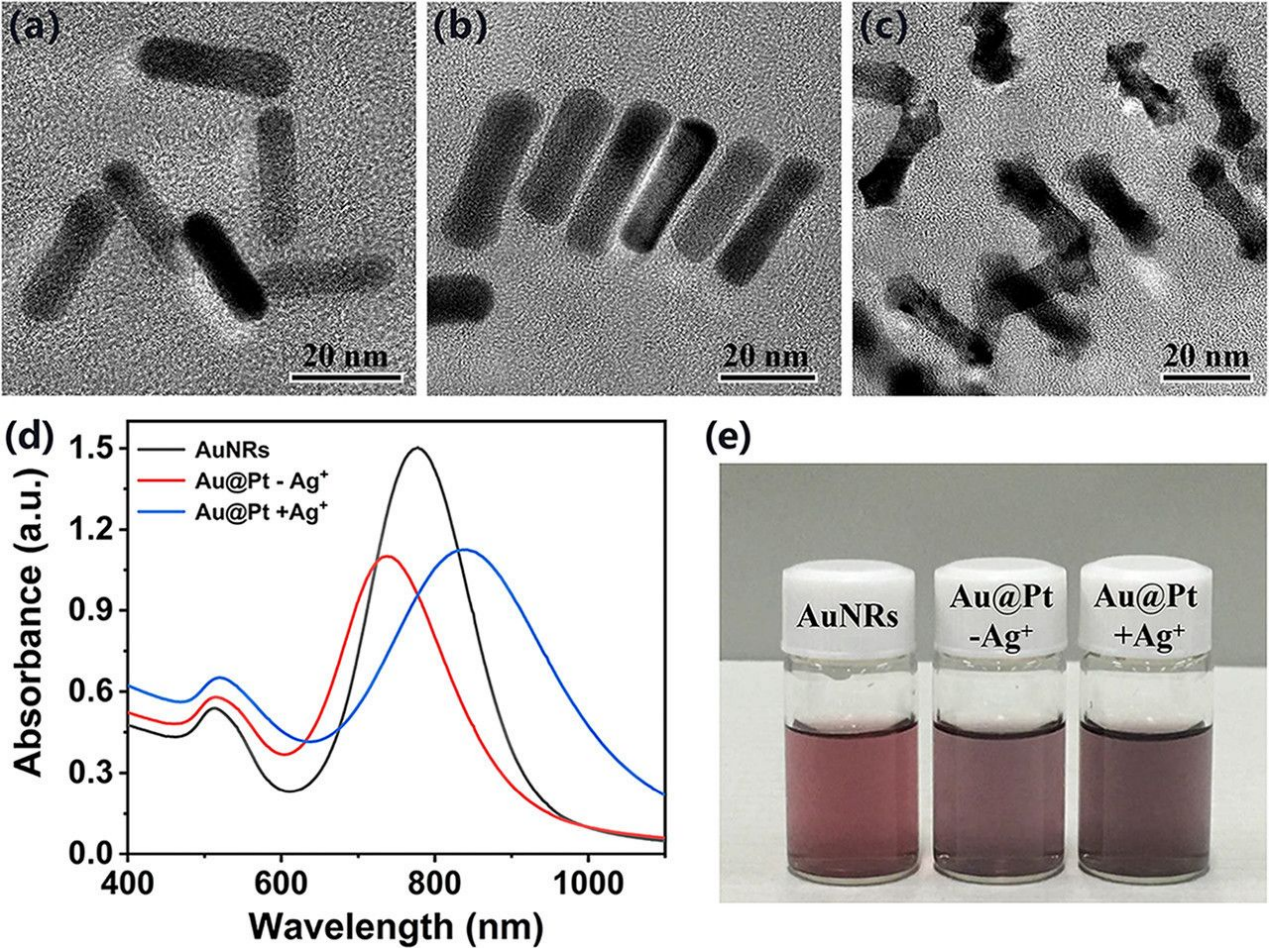
TEM images of (a) AuNRs, (b) Au@Pt−Ag^+^, and (c) Au@Pt+Ag^+^. (d) Absorption spectra of AuNRs (black), Au@Pt−Ag^+^ (red), and Au@Pt+Ag^+^ (blue). (e) Dispersibility of AuNRs, Au@Pt−Ag^+^, and Au@Pt+Ag^+^ in water. Annotation: Au@Pt−Ag^+^ and Au@Pt+Ag^+^ represent Au@Pt prepared in the absence and presence of Ag^+^, respectively.

The synthesis of AuNRs was carried out by a seedless-growth method with some modifications of reagent volume in the reaction solution. Figure 1a shows the morphology and size of as-prepared AuNRs characterized by TEM. According to the statistical data obtained from TEM images, the average length of the prepared particles is 26.8 ± 3.2 nm, the average width is 6.8 ± 1.1 nm, and the aspect ratio is close to 3.9 (all dimensions in this paper were calculated by counting 100 nanoparticles from TEM images). AuNRs with this aspect ratio showed a longitudinal LSPR peak at around 778 nm, as shown in Figure 1d and Table 1.

**Table 1:** Dimensions and optical properties of AuNRs, Au@Pt−Ag^+^, and Au@Pt+Ag^+^.

Nanorods	Length (*L*, nm)	Middle width (*W*_m_, nm)	Tip width (*W*_t_, nm)	Aspect ratio (*L*/*W*_m_)	LSPR λ (nm)

AuNRs	26.8 ± 3.2	6.8 ± 1.1	6.8 ± 1.1	3.9	778
Au@Pt−Ag^+^	32.6 ± 2.9	9.4 ± 1.4	10.1 ± 0.7	3.5	738
Au@Pt+Ag^+^	29.2 ± 2.8	6.8 ± 0.6	10.6 ± 1.5	4.3	836

### Effect of Ag^+^ on the preparation of Au@Pt NRs

In this section, we first discuss the anisotropic growth of Pt-coated AuNRs with or without Ag^+^ in the reaction solution. Then, we investigate the effect of the Ag^+^ concentration on the morphology and optical properties of the synthesized Au@Pt NRs. Regarding the growth of Pt on the surface of AuNRs, previous reports [28] indicated that Pt deposition on AuNRs occurs in two different modes, depending on the presence or absence of Ag^+^ in the reaction solution. In the initial experiments, absence or presence of Ag^+^ in the solution were achieved by centrifugation or keeping the Ag^+^ ions in freshly prepared AuNRs solutions, respectively.

When Ag^+^ was removed, CTAB and K_2_PtCl_4_ were added to the cleaned AuNRs solution with an optical density (OD) of 1.5. Please note that the AgNO_3_ solution was not added to the mixture, then ascorbic acid was added as reducing agent. The reaction solution was heated to 40 °C and kept at this temperature for 3 h. As shown in Figure 1b, the length of the synthesized products (Au@Pt−Ag^+^) increased to 32.6 ± 2.9 nm, the width increased to 9.4 ± 1.4 nm, and the aspect ratio was about 3.5. The results indicated that a complete and homogeneous Pt coating of about 2.5 nm thickness is formed on the surface of the AuNRs. In addition, the longitudinal LSPR peak of Au@Pt−Ag^+^ is blueshifted to 738 nm, due to the changes in geometric shape and aspect ratio, as shown in Figure 1d and Table 1. Grzelczak et al. proposed an electric field-directed mechanism to explain this phenomenon [27]. The concentration of CTAB in the reaction solution was 8.8 mM. This is much higher than the critical micelle constant (1.04–1.41 mM) of CTAB at this temperature [29–31], thus CTAB micelles were formed in the solution. PtCl_4_^2−^ is complexed with CTAB micelles and then gets reduced at the surface of AuNRs by ascorbic acid. During the reaction, a relatively obvious Pt shell formed gradually.

In sharp contrast, in the presence of Ag^+^, it is apparent that Pt grows mainly at the tip of the AuNRs, as shown in Figure 1c. This deposition pattern resulted in an anisotropic growth of the products (Au@Pt+Ag^+^), increasing the length to 29.2 ± 2.8 nm. The width at the middle of 6.8 ± 0.6 nm did not increase significantly. However, the width of the tips increased to 10.6 ± 1.5 nm. This is noticeable larger than the width at the middle and eventually led to dumbbell-like Au@Pt NRs. Furthermore, Pt deposition on the tip of AuNRs increases the aspect ratio, thereby redshifting the longitudinal LSPR peak to 836 nm, as shown in Figure 1d. These data are summarized in Table 1. In particular, the increased dephasing of the plasmons at the Au@Pt+Ag^+^ interface broadens the longitudinal LSPR band [32]. For the formation of dumbbell-like Au@Pt NRs, two main mechanisms have been proposed analogous to the formation of AuNRs. One reason is underpotential deposition (UPD) of Ag^+^, which gets reduced to Ag^0^ at the surface of the AuNRs with a lower surface potential than the reduction potential of Ag^+^ [27]. Electrochemical and crystallographic studies have shown that deposition of Ag^+^ on the side of the AuNRs (i.e., {110} facets) should be faster than on the tip (i.e., {100} facets) [33]. Another reason is the formation of strong silver bromide complexes (in the form of either Ag(I)-Br^−^-CTA^+^ or simply as AgBr_2_^−^), which act as face-specific capping agents on the lateral facets of the AuNRs [34–39]. Considering the above two mechanisms, it can be stated that when the PtCl_4_^2−^-CTAB complexes approach the surface of AuNRs, the reduction at the side is hindered, so they are preferentially deposited at the tip.

Regardless of the mode of Pt deposition on the surface of AuNRs, the color of the reaction solution changed from initial fuchsia to final purple gray, indicative of successful Pt coverage on the AuNRs [40]. The Au@Pt NRs dispersed well in water without notable aggregation, as shown in Figure 1e. In this paper, we do not conduct a detailed study on the action mechanism of Ag^+^ in the process of Au@Pt NRs synthesis, but rather focus on the practical consequences for the synthesized product. Next, we study the effects of different concentrations of Ag^+^ on the morphology and optical properties of the synthesized Au@Pt NRs.

**Figure 2:**
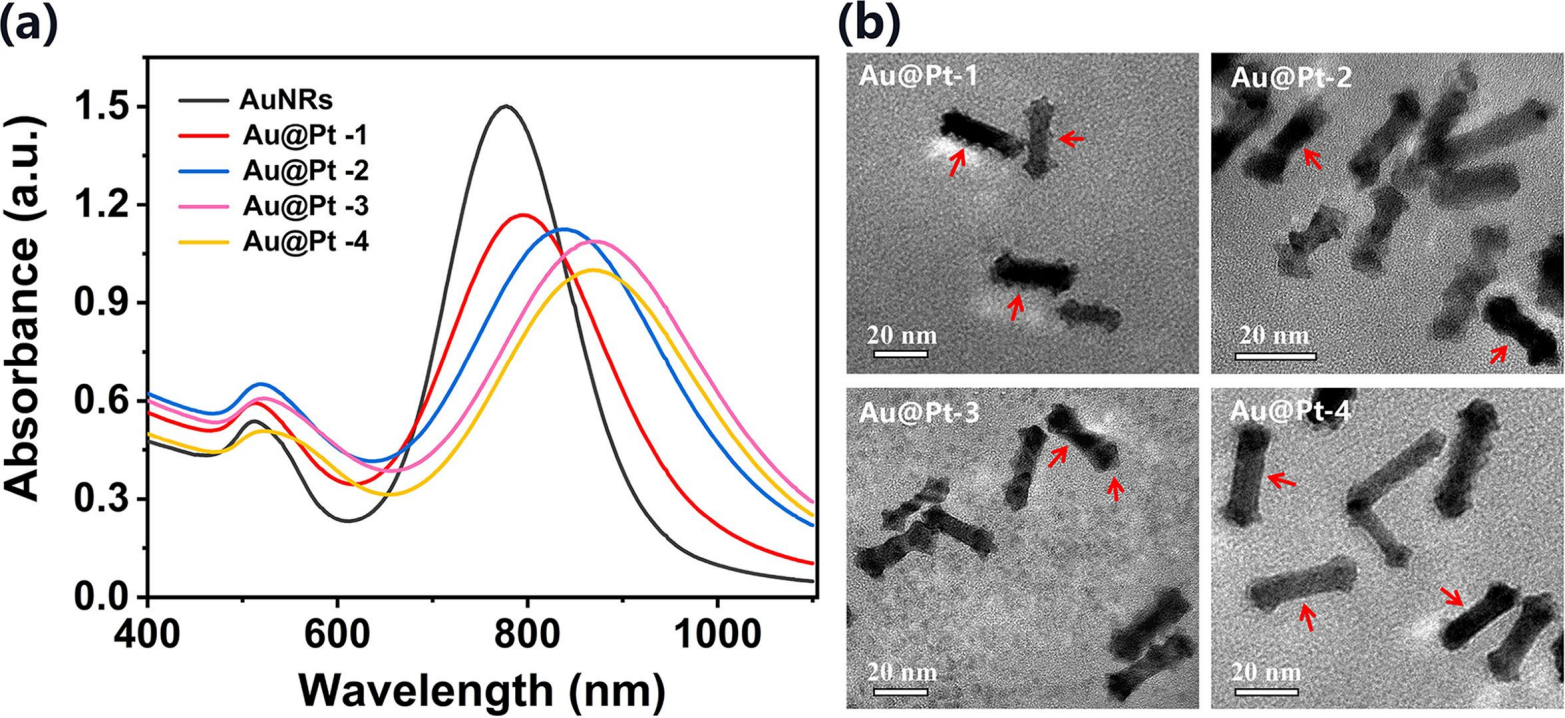
(a) Absorption spectra of AuNRs and of Au@Pt NRs synthesized with different concentrations of Ag^+^. (b) Corresponding TEM images of different Au@Pt NRs. Annotation: Au@Pt-1, Au@Pt-2, Au@Pt-3 and Au@Pt-4 represent the Au@Pt NRs prepared with different concentrations of added AgNO_3_: 5, 10, 20, and 40 mM, respectively.

In the AuNRs solution freshly obtained after the reaction, the concentration of excessive unreacted Ag^+^ cannot be clearly determined. The prepared AuNRs were washed three times by centrifugation and resuspension in deionized water, so that there was no excess Ag^+^ in the final AuNRs solution. After adding 5 μL AgNO_3_ solution of 5, 10, 20, and 40 mM concentration during the Pt deposition reaction, the longitudinal LSPR peaks of the obtained Au@Pt NRs (abbreviated as Au@Pt-1, Au@Pt-2, Au@Pt-3, and Au@Pt-4) were redshifted to 796, 836, 868, and 870 nm, respectively, as shown in Figure 2a. The obvious change of absorption spectra reveals non-negligible differences in the morphology of the different Au@Pt NRs. This hypothesis was further confirmed by TEM measurements, which are summarized in Figure 2b. From these TEM images we can see that Pt reduction occurred on the both side and tip of the AuNRs when the Ag^+^ content (5 μL of 5 mM AgNO_3_) is extremely small in the reaction solution. Different from Au@Pt−Ag^+^, the Pt coating on the side of AuNRs is not flat and smooth, but is rather a granular heterogeneous coating, as shown in Figure 2b Au@Pt-1 (red arrows). Interestingly, with the increase of Ag^+^ in the solution, it can be seen from Figure 2b Au@Pt-2 to Au@Pt-4 (red arrows) that the sides of AuNRs gradually become smoother, while the width at the tip increased further. This phenomenon may indicate that the increase in the quantity of Ag^+^ leads to the formation of more Ag^0^ or silver bromide complexes on the side of the AuNRs, which prevents the reduction of Pt to a greater extent, and Pt is mainly deposited on the tip at last.

Above all, variations of the LSPR bands would serve as a preferred indication of the Pt deposition on the AuNRs. Pt reduction leads to a stable, continuous, and significant redshift in the longitudinal LSPR peak as the Ag^+^ concentration increases. This suggests that the selective synthesis of Au@Pt NRs within a specific longitudinal LSPR peak range can be obtained by adjusting the concentration of Ag^+^ in the reaction solution.

### Effect of K_2_PtCl_4_ on the preparation of Au@Pt

**Figure 3:**
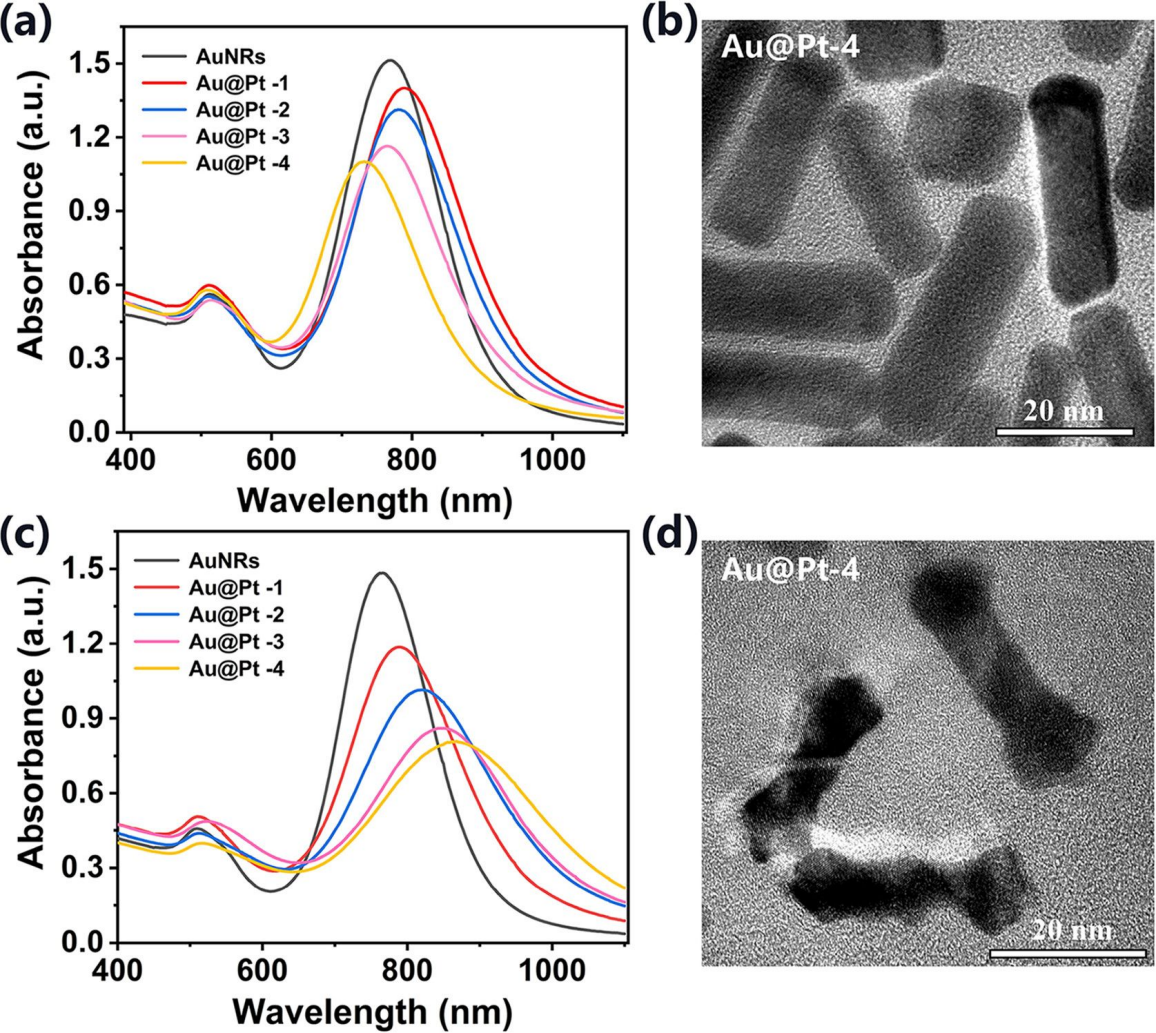
Absorption spectra of AuNRs and of different Au@Pt NRs synthesized in (a) absence and (c) presence of Ag^+^. (b) and (d) are TEM images of Au@Pt-4 in (a) and (c), respectively. Annotation: Au@Pt-1, Au@Pt-2, Au@Pt-3, and Au@Pt-4 represent the Au@Pt NRs prepared with different concentrations of added K_2_PtCl_4_: 5, 10, 20, and 40 mM, respectively.

Another important issue in the synthesis experiment of Au@Pt NRs is related to the influence of the amount of Pt in the reaction solution on the morphology and structure of the products. Therefore, in the absence and presence of Ag^+^, K_2_PtCl_4_ solutions of different concentration were added to the reaction solution for the deposition of Pt on AuNRs. In the absence of Ag^+^, it can be seen from the absorption spectra that when the concentrations of added K_2_PtCl_4_ were small (5 and 10 mM), the LSPR peaks of the products had a small redshift relative to the bare AuNRs, as shown in Figure 3a Au@Pt-1 and Au@Pt-2. Similar to the formation mechanism of AuNRs, the curvature of the tip is larger than that of the side, resulting in a preferential deposition of Pt on the tip of the AuNRs. This is also why the tip of the Au@Pt NRs observed in Figure 1b and Figure 3b is slightly wider than the middle. However, as the concentrations of added K_2_PtCl_4_ in the reaction solution continued to increase (20 and 40 mM), the LSPR peaks of the products obviously blueshifted, as shown in curve Au@Pt-3 and Au@Pt-4 in Figure 3a. We hypothesize that with the increase of K_2_PtCl_4_ content, the collision frequency between PtCl_4_^2−^-CTAB micelles and AuNRs also increased, and eventually, Pt smoothly covers the surface of AuNRs and forms a thicker Pt coating. The absorption spectra and the TEM image of the product Au@Pt-4 confirmed our conjecture, as shown in Figure 3a and 3b. The LSPR peak of Au@Pt-4 is at a wavelength of 728 nm, the length is about 34.6 ± 2.4 nm, the width is about 10.5 ± 1.2 nm, the aspect ratio is about 3.3, and the thickness of the Pt shell on the surface of the AuNRs is about 3.8 nm, which is thicker than the Pt coating of the sample Au@Pt−Ag^+^ (the concentration of K_2_PtCl_4_ added is 30 mM) in Figure 1b.

In the presence of Ag^+^, which can be clearly seen from Figure 3c, the LSPR peaks continuously redshifted with increasing amount of K_2_PtCl_4_, indicating that the aspect ratio of the NRs increased. Further from the obtained TEM image of the product Au@Pt-4, it was statistically concluded that the length is about 30.4 ± 1.9 nm and the width of the middle is about 6.8 ± 1.2 nm. In particular, the width of the tip is about 11.5 ± 1.7 nm, which is wider than the tip width of the sample Au@Pt+Ag^+^ (the concentration of K_2_PtCl_4_ added is 30 mM) in Figure 1c. The results show that more Pt was deposited at the tip of AuNRs, which is consistent with the absorption spectra.

**Figure 4:**
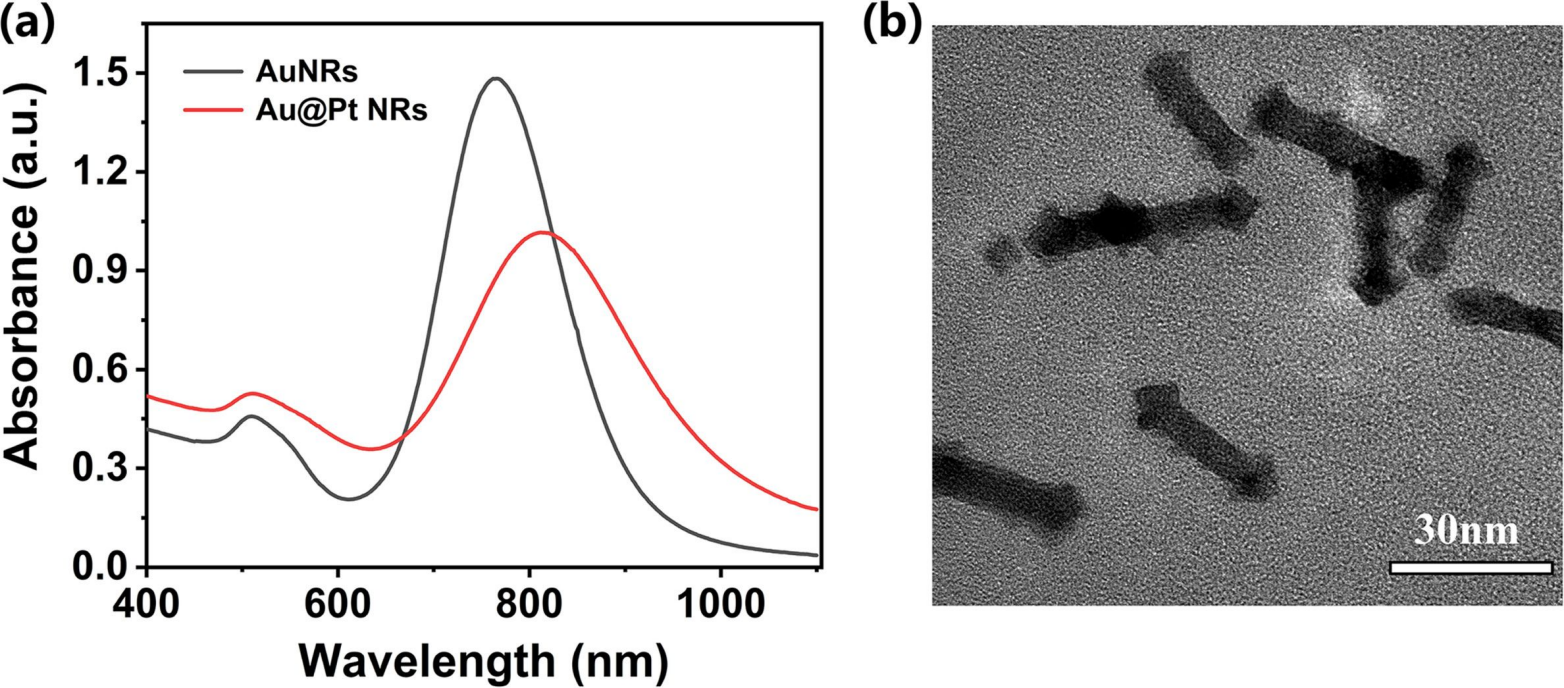
(a) Absorption spectra of AuNRs and of Au@Pt NRs prepared under optimal conditions. (b) TEM image of Au@Pt NRs.

Considering that the aim of the work is to obtain Au@Pt NRs with high PCE, a LSPR peak of the Au@Pt NRs close to 808 nm is desirable. The optimized final dosage of each reagent and the experimental procedure are described in detail in part Experimental. What we want to emphasize here is that the synthesis only takes 3 h at 40 °C without the need for elaborate instrumentation. The absorption spectrum of the optimized Au@Pt NRs is shown in Figure 4a. The LSPR peak is located at a wavelength of 812 nm, which is very satisfactory for the photothermal effect under laser irradiation with 808 nm. The corresponding micrograph is shown in Figure 4b.

### Photothermal effect

**Figure 5:**
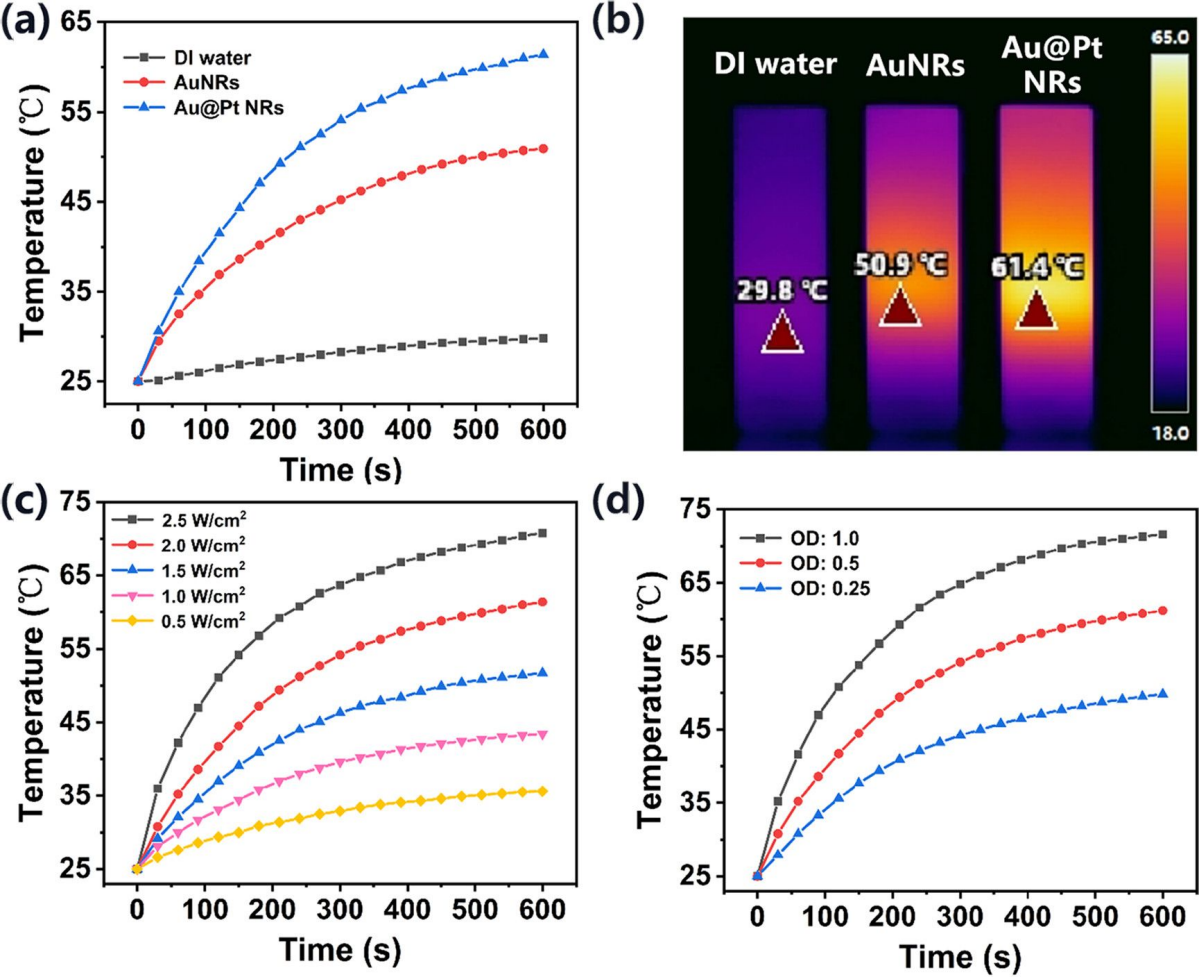
(a) Temperature elevation curves of deionized water (black), AuNRs solution (OD = 0.5, red) and Au@Pt NRs solution (OD = 0.5, blue) upon exposure to 808 nm laser excitation (2 W/cm^2^, 10 min). (b) Infrared thermal images of the three solutions after 600 s of laser exposure. (c) Temperature elevation curves of Au@Pt NRs solution (OD = 0.5) under different laser power densities (10 min). (d) Temperature elevation curves of Au@Pt NRs solution with different OD under 808 nm laser irradiation (2.0 W/cm^2^, 10 min).

To test the efficiency of AuNRs and the optimized Au@Pt NRs as PTAs, their PTE under 808 nm laser irradiation was systematically studied. The temperature changes were recorded using an infrared thermal camera, as shown in Figure 5a,b. After 10 min of 808 nm laser irradiation (2 W/cm^2^), the temperature of the AuNRs solution increased from the initial 25 °C to 50.9 °C, while the temperature of deionized water rose by only 5 °C. In comparison, the temperature of the Au@Pt solution reached about 54.1 °C after only 5 min of irradiation and quickly reached a peak of 61.4 °C, which demonstrated significant enhanced photothermal heating. The Au@Pt NRs solution exhibits a stronger thermal response than AuNRs with the same amount of Au. Due to the fact that the LSPR of Au@Pt NRs is closer to 808 nm and has a wider band, it absorbs more photon energy and releases more heat. Next, to explore the influence of laser power on the photothermal effect, the Au@Pt NRs solution was exposed to 808 nm laser irradiation with different power density values. It can be clearly observed from Figure 5c that as the power density increases, the temperature also increases. Similarly, the absorbance of the Au@Pt NRs solution is also positively correlated with the temperature, as shown in Figure 5d.

In addition, we measured the PCE (η) of AuNRs and Au@Pt NRs according to the model proposed by Roper et al. [41]:

[1]
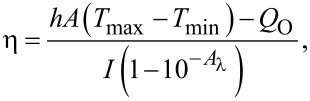


where *h* is the heat-transfer coefficient, *A* is the irradiated surface area of the cuvette, *T*_max_ and *T*_min_ are the maximum and minimum steady-state temperatures of the solution, respectively, *Q*_O_ is the heat dissipated from light absorbed by the cuvette and the deionized water, *I* is the incident laser power (2 W), and *A*_λ_ is the absorbance of the solution at 808 nm.

**Figure 6:**
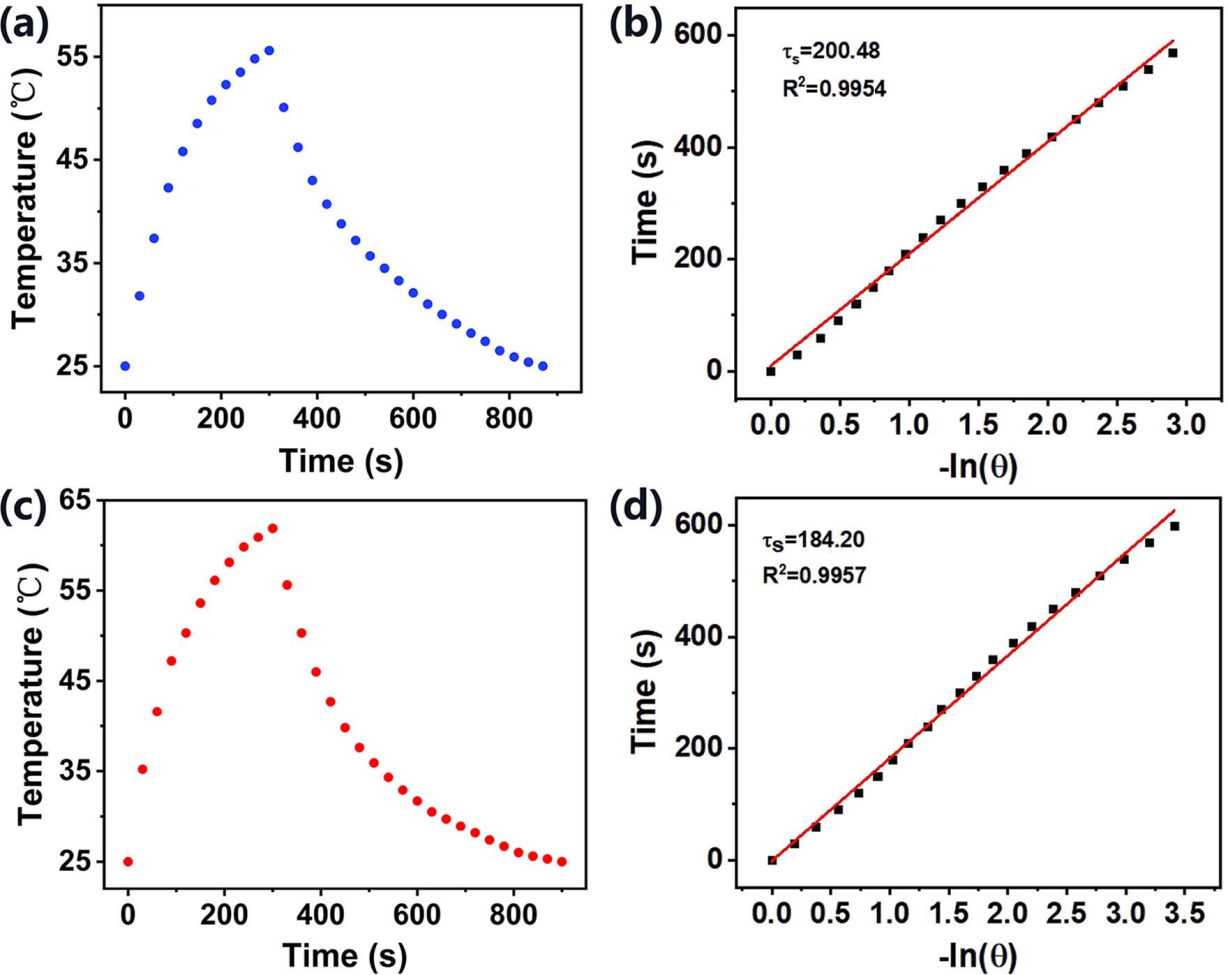
Heating and cooling curves of (a) AuNRs and (c) Au@Pt NRs under 808 nm laser irradiation. Linear fit of the time vs −ln θ obtained from the cooling period of the (b) AuNRs and (d) Au@Pt NRs heating experiments.

*Q*_O_ was measured in an independent experiment. In our system, it was calculated as 96.58 mW by using a cuvette containing 2.0 mL of deionized water. The value of *hA* can be determined by considering the linear time data from the cooling period vs −ln θ as shown in Figure 6b,d. It can be calculated using the following equations:

[2] $$hA=\frac{mC_{\text{P}}}{\tau_{\text{s}}},$$

[3]
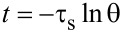


[4] $$\theta =\frac{T-T_{\min}}{T_{\max}-T_{\min}},$$

where *m* and *C*_P_ are mass (1.8 g) and specific heat capacity (pure water: 4.2 J/(g·K)), τ_s_ is the system time constant, *T* is the temperature of the solution during cooling after the laser was turned off.

Entering the values of *Q*_O_ and *hA* into Equation 1, the calculated PCE (η) of Au@Pt NRs was determined to be 78.77%, which is much higher than the calculated PCE (η) of AuNRs of 57.33%. In summary, the Au@Pt NRs exhibit a stronger photothermal effect than AuNRs.

**Figure 7:**
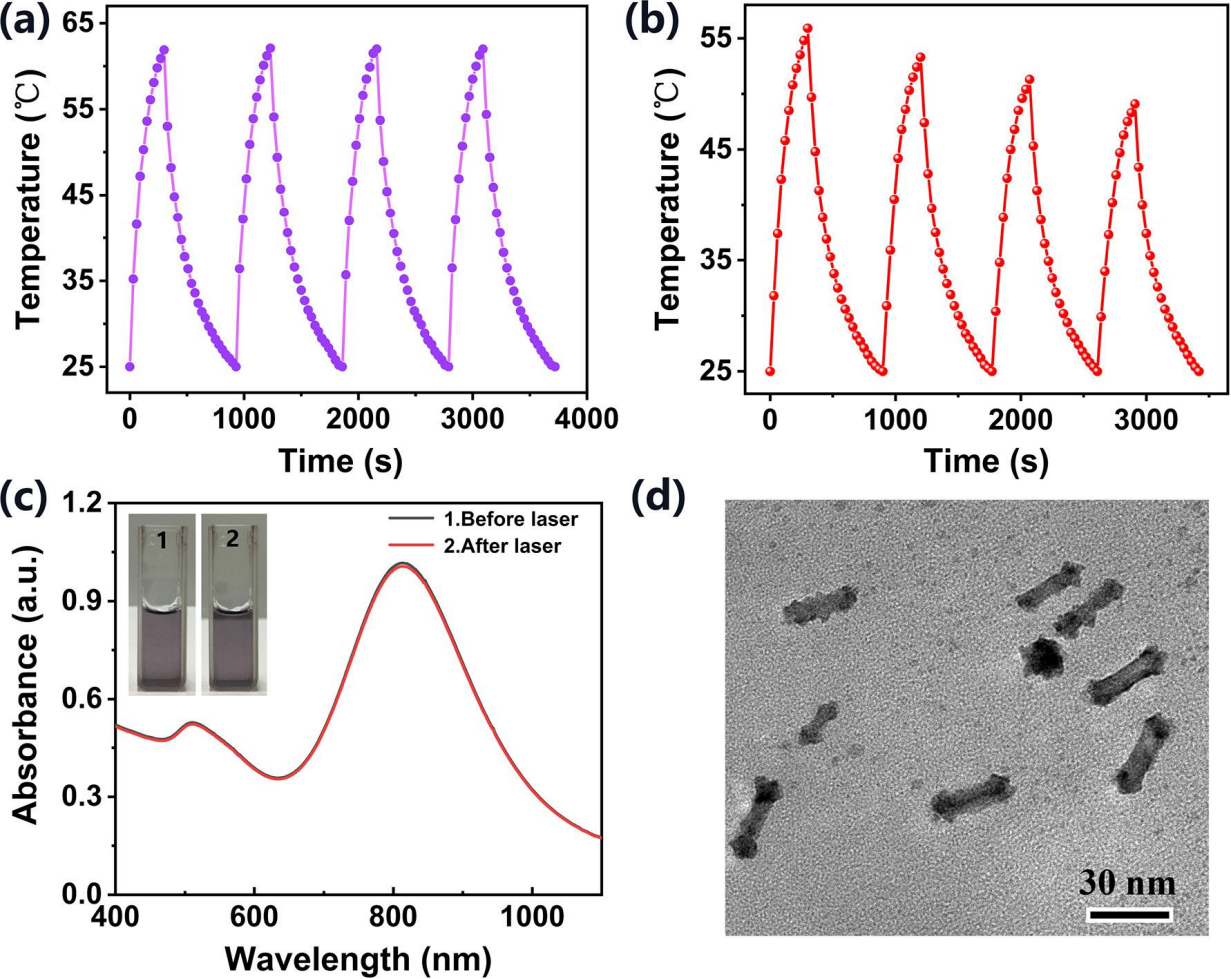
Temperature change curve of (a) Au@Pt NPs solution and (b) AuNRs solution during four on/off cycles of irradiation. (c) Absorption spectra and photographs of Au@Pt NPs solution before and after cycled laser irradiation. (d) TEM image of Au@Pt NPs after cycled laser irradiation.

We next evaluated the photothermal stability of the Au@Pt NRs, which is a very important property of PTA materials. First, the temperature changes of Au@Pt NRs solution were observed under cycled 808 nm laser irradiation. As shown in Figure 7a, the four temperature peaks of Au@Pt NRs solution showed only very little changes, and the peaks raised above 60 °C in each “on” cycle, which shows great photothermal stability and reusability. In contrast, the laser irradiation stability of AuNRs is not as satisfactory. As shown in Figure 7b, each irradiation reduces the peak temperature of the next irradiation. After four cycles of irradiation, the maximum temperature decreased from 55.9 to 49.1 °C. Consistently with previously reported results, AuNRs melt into Au nanospheres under prolonged or repeated 808 nm laser irradiation, and their PCE decreases as the LSPR peak moves away from the wavelength of the laser [15,42–43]. Besides, as shown in Figure 7c, the absorption spectrum of the sample remained basically unchanged after four cycles, indicating that the synthesized Au@Pt NRs were not damaged by the laser and their original optical properties were retained. The photograph of the Au@Pt NRs solution also reflects that it has the same great water dispersibility before and after laser irradiation. Finally, the TEM image in Figure 7d visually confirmed that Au@Pt NRs still maintained their integrity after four laser irradiations, showing almost no change in morphology and size distribution.

## Conclusion

We report a simple method for preparing dumbbell-like Au@Pt NRs under mild conditions. By controlling the concentration of Ag^+^ and K_2_PtCl_4_ in the reaction solution, Au@Pt NRs were synthesized with different morphology and longitudinal LSPR peaks within a specific small range. In this paper, the Au@Pt NRs with a vertical LSPR peak close to 808 nm were synthesized. It was verified that the Au@Pt NRs have a higher PCE under 808 nm laser irradiation than other PTAs. Also, they exhibit excellent photothermal stability. Our work has paved the way for the controllable synthesis of Au@Pt NRs with specific optical properties and stable photothermal performance. The Au@Pt NRs are capable of acting as a novel PTAs and show good application prospects in the photothermal therapy of tumors.

## Experimental

### Reagents

Gold(III) chloride trihydrate (HAuCl_4_·3H_2_O), potassium tetrachloroplatinate(II) (K_2_PtCl_4_), silver nitrate (AgNO_3_), cetyltrimethylammonium bromide (CTAB), ascorbic acid, and sodium borohydride (NaBH_4_) were purchased from Sinopharm Chemical Reagent Co. Ltd. (Taiyuan, China). Deionized water was used in all experiments.

### Instruments

The absorption spectra were measured on a UV–vis–NIR spectrophotometer (UV-8000A, 190–1200 nm, Metash, China). Transmission electron microscopy (TEM) images, morphology, and size of nanoparticles were obtained with a transmission electron microscope (JEOL 2100F, 200 kV, Hitachi, Japan). In the photothermal experiment, an 808 nm CW laser (MDL-III-808 nm, CNI, China) was used to irradiate the nanoparticle solutions, and temperature changes of all samples were recorded with an infrared thermal camera (225s, Fotric, China).

### Preparation of AuNRs

AuNRs were synthesized following the seedless-growth technique by Ali et al. with minor modifications [44]. Briefly, at first, 208 μL of 24 mM HAuCl_4_ and 100 μL of 10 mM AgNO_3_ were mixed with 10 mL of 100 mM CTAB evenly in an Erlenmeyer flask, at which point the solution was yellow. Next, 55 μL of 100 mM ascorbic acid solution was added and stirred well, which formed the growth solution. The color of the growth solution rapidly changed from yellow to colorless. Finally, 15 μL of 1 mM NaBH_4_ solution (prepared with ice water) was added and stirred vigorously at room temperature, then the solution gradually turned lavender red. After remaining at a constant temperature of 30 °C for 6 h, the obtained AuNRs solution was centrifuged at 11,000 rpm for 25 min. The supernatant was removed and the remaining pellet was diluted in deionized water. The centrifugation and dilution operations were repeated for three times to ensure that there was no excess CTAB and Ag^+^ in the AuNRs solution. AuNRs with uniform particle size and good dispersion were obtained.

### Platinum coating of AuNRs

Using a slightly modified experimental method of Grzelczak et al. [27], Pt was deposited on the tips of the AuNRs in the presence of Ag^+^. The cleaned AuNRs were re-dispersed in deionized water, 1 mL CTAB (100 mM) and 3 μL AgNO_3_ (10 mM) were added to 10 mL AuNRs solution with an OD of 1.5. After that, 100 μL of 10 mM K_2_PtCl_4_ and 200 μL of 100 mM ascorbic acid solution were added to the mixture at 40 °C, and the solution was kept at 40 °C for 3 h to ensure effective reduction of Pt. Au@Pt NRs were purified by centrifugation at 11,000 rpm for 10 min and finally resuspended in deionized water.

### Photothermal efficiency evaluation

To compare the photothermal effect of the as-synthesized AuNRs and Au@Pt NRs, the two NRs solutions were diluted in deionized water so that they had the same OD of 0.5. Then, 2 mL AuNRs solution and 2 mL Au@Pt NRs solution were added to two quartz cuvettes and exposed to an 808 nm cw laser at 2.0 W/cm^2^ for 10 min. At definite time intervals, the temperature changes were recorded with the infrared thermal camera. For comparison, the temperature change of 2 mL deionized water was also measured. The initial temperature of all solutions was 25 °C.

In addition, to evaluate the photothermal stability of Au@Pt NRs, an on/off cycle irradiation experiment was performed. The Au@Pt NRs solution at an initial temperature of 25 °C was irradiated by 808 nm CW laser for 5 min, and then the laser was turned off immediately. When the solution had cooled down naturally to the initial temperature of 25 °C, the laser was turned on again. This process was repeated for four cycles and the temperature variation was recorded. Also, before and after the cycle irradiation, the absorption spectra and the morphology of Au@Pt NRs were measured by spectrophotometry and TEM.

### Statistical analysis

Results are expressed as the average ± standard deviation for each size of more than 100 nanorods from at least six different TEM images. Statistical significance was calculated using a *t*-test or analysis of variance (ANOVA) calculator (GraphPad Software, Inc.) and *p* < 0.05.
